# Optimized Signal Peptide for Secretory Expression of Human Recombinant Somatropin in *E. coli*

**DOI:** 10.34172/apb.2023.037

**Published:** 2022-04-04

**Authors:** Zeynab Ahmadi, Safar Farajnia, Davoud Farajzadeh, Naser Pouladi, Neda Pourvatan, Mohammad Karbalaeimahdi, Fahime Shayegh, Maryam Arya

**Affiliations:** ^1^Department of Biology, Faculty of Basic Sciences, Azarbaijan Shahid Madani University, Tabriz, Iran.; ^2^Drug Applied Research Center, Tabriz University of Medical Science, Tabriz, Iran.; ^3^Biotechnology Research Center, Tabriz University of Medical Science, Tabriz, Iran.; ^4^Department of Molecular Biology and Cancer Research, Azarbaijan Shahid Madani University, Tabriz, Iran.; ^5^Immunology Research Center, Tabriz University of Medical Science, Tabriz, Iran.

**Keywords:** Human somatropin, Signal peptide, E. coli, Secretary expression

## Abstract

***Purpose:*** The human somatropin is a single-chain polypeptide with a pivotal role in various biological processes. Although *Escherichia coli* is considered as a preferred host for the production of human somatropin, the high expression of this protein in *E. coli* results in the accumulation of protein as inclusion bodies. Periplasmic expression using signal peptides could be used to overcome the formation of inclusion bodies; still, the efficiency of each of the signal peptides in periplasmic transportation is varied and often is protein specific. The present study aimed to use *in silico* analysis to identify an appropriate signal peptide for the periplasmic expression of human somatropin in *E. coli*.

***Methods:*** A library containing 90 prokaryotic and eukaryotic signal peptides were collected from the signal peptide database, and each signal’s characteristics and efficiency in connection with the target protein were analyzed by different software. The prediction of the secretory pathway and the cleavage position was determined by the signalP5 server. Physicochemical properties, including molecular weight, instability index, gravity, and aliphatic index, were investigated by ProtParam software.

***Results:*** The results of the present study showed that among all the signal peptides studied, five signal peptides ynfB, sfaS, lolA, glnH, and malE displayed high scores for periplasmic expression of human somatropin in *E. coli*, respectively.

***Conclusion:*** In conclusion, the results indicated that in-silico analysis could be used for the identification of suitable signal peptides for the periplasmic expression of proteins. Further laboratory studies can evaluate the accuracy of the results of *in silico* analysis.

## Introduction

 Human somatropin is a non-glycosylated single-chain polypeptide comprising of 191 amino acids, with a molecular mass of 22.1 kDa.^1^ Somatropin belongs to the somatotropin/prolactin family, which plays a significant role in growth control through stimulating various tissues, mainly the liver, to secrete insulin-like growth factor 1 (IGF-1). Besides, it is responsible for the differentiation and proliferation of myoblasts, the uptake of amino acids, and proteins’ production in muscles and other tissues.^2^

 Advantages such as easy genetic manipulation, low-cost media, and short culturing time have led to the use of *Escherichia coli *as the most suitable expression system for the production of many recombinant proteins.^3^ However, high level expression of recombinant proteins in *E. coli* often give rise to aggregated protein molecules, known as inclusion bodies.^4^ Therefore, recombinant proteins’ purification encounters significant challenges, involving isolation from the cells, unfolding, refolding, and purification to produce the bioactive proteins. Various strategies have been used to overcome this problem include secretary expression by targeting the protein into the periplasmic space by an N-terminal signal peptide.^5^

 Sec, SRP, and TAT are major protein secretion pathways used by prokaryotes by which proteins direct into the periplasm or extracellular space according to their signal peptides (signal peptides).^6^ Therefore, selecting an appropriate signal peptide is an essential parameter in the secretory expression of recombinant proteins.^7^ Several studies have shown that the function of signal peptides is protein-specific, and there is no unique ideal signal peptide for secretary expression of all proteins.^8^ A conventional method for selecting a signal peptide for a given protein is trial and error, which is labor-intensive and time-consuming. Recently various bioinformatics programs have been developed for the analysis of the efficiency of different signal peptides, which include signalP4.1, ProtParam, SOLpro, ProtCompB, and signalP5.0. The advantages of using a bioinformatics program before starting an experimental study are reducing costs and increasing the accuracy and validity of experimental research.^9^

 Secretory expression of recombinant proteins, particularly pharmaceutical proteins, in *E. coli* has many advantages. Targeting a recombinant protein to the periplasmic space or the extracellular medium, in addition to reducing costs, facilitates downstream processing, compared to the cytosolic production.^10^

 The purpose of the present study was to *in silico* analysis of various signal peptides for secretary expression of somatropin using different bioinformatic programs.

## Materials and Methods

###  Signal peptide sequences 

 In this research, sequences of 90 different signal peptides were collected from the Signal Sequence database at http://www.signalpeptide.de/ (Table 1) and used for further analyses.

**Table 1:** The list of signal peptides was evaluated in this study

**Full name**	**Signal peptide**	**Length**	**Source**	**Accession number**	**Amino acid sequence**
Periplasmic appA protein	appA	22	*Escherichia coli* (strain K12)	P07102	MKAILIPFLSLLIPLTPQSAFA
Cytochrome c-type biogenesis protein	ccmH	18	*Escherichia coli* (strain K12)	P0ABM9	MRFLLGVLMLMISGSALA
Protein cexE	cexE	19	*Escherichia coli*	A2TJI4	MKKYILGVILAMGSLSAIA
Thiosulfate-binding protein	cysP	25	*Escherichia coli* (strain K12)	P16700	MAVNLLKKNSLALVASLLLAGHVQA
Drhemagglutinin structural subunit	draA	21	*Escherichia coli*	P24093	MKKLAIMAAASMVFAVSSAHA
Thiol:disulfide interchange protein dsbD	dsbD	19	*Escherichia coli *(strain K12)	P36655	MAQRIFTLILLLCSTSVFA
Thiol:disulfide interchange protein dsbG	dsbG	17	*Escherichia coli* (strain K12)	P77202	MLKKILLLALLPAIAFA
K88 fimbrail protein AD	faeG	21	*Escherichia coli*	P14191	MKKTLIALAIAASAASGMAHA
Iron(III) dicitrate-binding periplasmic protein	fecB	21	*Escherichia coli *(strain K12)	P15028	MLAFIRFLFAGLLLVISHAFA
F107 fimbrail protein	fedA	21	*Escherichia coli*	P25394	MKRLVFISFVALSMTAGSAMA
F41 fimbrail protein	FimF41a	22	*Escherichia coli*	P11900	MKKTLIALAVAASAAVSGSVMA
Flagellar P-ring protein	flgI	20	*Escherichia coli *O1:K1 / APEC	A1A9X5	MVIKFLSALILLLVTTAAQA
Protein transport protein hofQ	hofQ	18	*Escherichia coli *(strain K12)	P34749	MKQWIAALLLMLIPGVQA
Outer-membrane lipoprotein carrier protein	lolA	21	*Escherichia coli* (strain K12)	P61316	MKKIAITCALLSSLVASSVWA
Lipopolysaccharide export system protein lptA	lptA	27	*Escherichia coli* (strain K12)	P0ADV1	MKFKTNKLSLNLVLASSLLAASIPAFA
Maltose-binding periplasmic protein	malE	26	*Escherichia coli* (strain K12)	P0AEX9	MKIKTGARILALSALTTMMFSASALA
Penicillin-insensitive murein endopeptidase	mepA	19	*Escherichia coli* O157:H7	Q8XCQ5	MNKTAIALLALLASSVSLA
Nickel-binding periplasmic protein	nikA	22	*Escherichia coli* (strain K12)	P33590	MLSTLRRTLFALLACASFIVHA
Cytochrome c-552	nrfA	26	*Escherichia coli* (strain K12	P0ABK9	MTRIKINARRIFSLLIPFFFFTSVHA
Outer membrane protein A	ompA	21	*Escherichia coli* (strain K12	P0A910	MKKTAIAIAVALAGFATVAQA
Outer membrane protease ompP	ompP	23	*Escherichia coli* (strain K12)	P34210	MQTKLLAIMLAAPVVFSSQEASA
Outer membrane protein W	ompW	21	*Escherichia coli* (strain K12)	P0A915	MKKLTVAALAVTTLLSGSAFA
Fimbrial adapter papK	papK	21	*Escherichia coli*	P62532	MIKSTGALLLFAALSAGQAIA
D-alanyl-D-alanine endopeptidase	pbpG	25	*Escherichia coli* (strain K12)	P0AFI5	MPKFRVSLFSLALMLAVPFAPQAVA
pectate lyase B	PelB	22	Erwinia chrysanthemi	P04959	MKYLLPTAAAGLLLLAAQPAMA
Alkaline phosphatase	phoA	21	*Escherichia coli* (strain K12)	P00634	MKQSTIALALLPLLFTPVTKA
Outer membrane pore protein E	phoE	21	*Escherichia coli* (strain K12)	P02932	MKKSTLALVVMGIVASASVQA
Protein prsK	prsK	21	*Escherichia coli*	P42191	MIKSTGALLLFAALSAGQAMA
Phage shock protein E	pspE	19	*Escherichia coli* (strain K12)	P23857	MFKKGLLALALVFSLPVFA
Protease 3	ptrA	23	*Escherichia coli* (strain K12)	P05458	MPRSTWFKALLLLVALWAPLSQA
S-fimbrial adhesin protein	sfaS	22	*Escherichia coli* O6:K15:H31	P13430	MKLKAIILATGLINCIAFSAQA
Taurine-binding periplasmic protein	tauA	22	*Escherichia coli* (strain K12)	Q47537	MAISSRNTLLAALAFIAFQAQA
Thiamine-binding periplasmic protein	thiB	18	*Escherichia coli* (strain K12)	P31550	MLKKCLPLLLLCTAPVFA
Periplasmic protein torT	torT	18	*Escherichia coli* (strain K12)	P38683	MRVLLFLLLSLFMLPAFS
Trimethylamine-N-oxide reductase 1	TorA	39	*Escherichia coli* (strain K12)	P33225	MNNNDLFQASRRRFLAQLGGLTVAGMLGPSLLTPRRATA
sn-glycerol-3-phosphate-binding periplasmic protein ugpB	ugpB	23	*Escherichia coli* (strain K12)	P0AG80	MKPLHYTASALALGLALMGNAQA
D-xylose-binding periplasmic protein	xylF	23	*Escherichia coli* (strain K12)	P37387	MKIKNILLTLCTSLLLTNVAAHA
Uncharacterized protein yfeK	yfeK	19	*Escherichia coli* (strain K12)	Q47702	MKKIICLVITLLMTLPVYA
UPF0379 protein yhcN	yhcN	22	*Escherichia coli* (strain K12)	P64614	MKIKTTVAALSVLSVLSFGAFA
Uncharacterized protein yncJ	yncJ	22	*Escherichia coli* (strain K12)	P64459	MFTKALSVVLLTCALFSGQLMA
UPF0482 protein ynfB	ynfB	28	*Escherichia coli* (strain K12)	P76170	MKITLSKRIGLLAILLPCALALSTTVHA
Zinc resistance-associated protein	zraP	26	*Escherichia coli* (strain K12)	P0AAA9	MKRNTKIALVMMALSAMAMGSTSAFA
Beta-lactamase	ampC	19	*Escherichia coli* (strain K12)	P00811	MFKTTLCALLITASCSTFA
Heat-labile enterotoxin B chain	eltB	21	*Escherichia coli*	P13811	MNKVKFYVLFTALLSSLCAHG
Type-1 fimbrial protein, C chain	pilC	23	*Escherichia coli*	P62605	MKLKFISMAVFSALTLGVATNAS
Copper resistance protein B	pcoB	23	*Escherichia coli*	Q47453	MKRNLKAIPVLVAGLFTSQLSIA
Serine protease eatA	eatA	56	*Escherichia coli*	Q84GK0	MNKVFSLKYSFLAKGFIAVSELARRVSVKGKLKSASSIIISPITIAIVSYAPPSLA
Hemoglobin-binding protease hbp	HBP	52	*Escherichia coli*	O88093	MNRIYSLRYSAVARGFIAVSEFARKVHKSVRRLCFPVLLLIPVLFSAGSLA
Thiol:disulfide interchange protein dsbA	DsbA	19	*Escherichia coli* (strain K12)	POAEG4	MKKIWLALAGLVLAFSASA
Human G.H.	Hgh	26	*Homo sapiens*	P01241	MATGSRTSLL LAFGLLCLPWLQEGSA
Outer membrane protein C	OmpC	21	*Escherichia coli* (strain K12)	P06996	MKVKVLSLLVPALLVAGAANA
Heat-stable enterotoxin II	STII	23	*Escherichia coli*	P22542	MKKNIAFLLASMFVFSIATNAYA
L-asparaginase 2	ansB	22	*Escherichia coli* (strain K12)	P00805	MEFFKKTALAALVMGFSGAALA
Chaperone protein sfmC	sfmC	23	*Escherichia coli* (strain K12)	P77249	MMTKIKLLMLIIFYLIISASAHA
Outer membrane protein F	ompf	22	*Escherichia coli* (strain K12)	P02931	MMKRNILAVIVPALLVAGTANA
Protease 7	ompt	20	*Escherichia coli* (strain K12)	P09169	MRAKLLGIVLTTPIAISSFA
Major outer membrane lipoprotein	LPP	20	*Escherichia coli* (strain K12)	P69776	MKATKLVLGAVILGSTLLAG
Maltoporin	lamB	25	*Escherichia coli* (strain K12)	P02943	MMITLRKLPLAVAVAAGVMSAQAMA
Beta-lactamase TEM	bla	23	*Escherichia coli*	P62593	MSIQHFRVALIPFFAAFCLPVFA
D-galactose-binding periplasmic protein	mglB	23	*Escherichia coli* (strain K12)	P0AEE5	MNKKVLTLSAVMASMLFGAAAHA
Heat-stable enterotoxin ST-IA/ST-P	Sta1	19	*Escherichia coli*	P01559	MKKLMLAIFISVLSFPSFS
L-arabinose-binding periplasmic protein	araF	23	*Escherichia coli* (strain K12)	P02924	MHKFTKALAAIGLAAVMSQSAMA
Putative outer membrane porin protein	nmpc	23	*Escherichia coli* (strain K12)	P21420	MKKLTVAISAVAASVLMAMSAQA
Peptidyl-prolyl cis-trans isomerase A	ppiA	24	*Escherichia coli* (strain K12)	P0AFL3	MFKSTLAAMAAVFALSALSPAAMA
UPF0412 protein YaaI	yaaI	23	*Escherichia coli* (strain K12)	P28696	MKSVFTISASLAISLMLCCTAQA
Uncharacterized protein YhcF	yhcF	20	*Escherichia coli* (strain K12)	P45422	MNNVKLLIAGSAFFAMSAQA
Uncharacterized fimbrial-like protein YfcQ	yfcQ	18	*Escherichia coli* (strain K12)	P76500	MRKTFLTLLCVSSAIAHA
Iron uptake system component EfeO	EfeO	26	*Escherichia coli* (strain K12)	P0AB24	MTINFRRNALQLSVAALFSSAFMANA
Glutamine-binding periplasmic protein	glnH	22	*Escherichia coli* (strain K12)	P0AEQ3	MKSVLKVSLAALTLAFAVSSHA
Ribonuclease I	rna	23	*Escherichia coli* (strain K12)	P21338	MKAFWRNAALLAVSLLPFSSANA
Disulfide interchange protein DsbC	DsbC	20	*Escherichia coli* (strain K12)	P0AEG6	MKKGFMLFTLLAAFSGFAQA
D-ribose-binding periplasmic protein	rbsB	25	*Escherichia coli* (strain K12)	P02925	MNMKKLATLVSAVALSATVSANAMA
Cyclic di-GMP-binding protein	bcsB	25	*Escherichia coli* (strain K12)	P37652	MKRKLFWICAVAMGMSAFPSFMTQA
Threonine-rich inner membrane protein GfcA	gfcA	21	*Escherichia coli* (strain K12)	P75885	MKHKLSAILMAFMLTTPAAFA
Salivary acidic proline -rich phosphoprotein	PRH1	22	*Homo sapiens*	P81277	MKVLRAWLLCLLMLGLALRGAA
Liver -expressed antimicrobial peptide2	LEAP2	22	*Homo sapiens*	Q969E1	MWHLKLCAVLMIFLLLLGQIDG
Secreted protein C10orf99	C10orf99	24	*Homo sapiens*	Q6UWK7	MRLLVLSSLLCILLLCFSIFSTEG
Prolactin -releasing peptide	PRLH	22	*Homo sapiens*	P81277	MKVLRAWLLCLLMLGLALRGAA
Heparin sulfate proteoglycan core protein	HSPG2	21	*Homo sapiens*	P98160	MGWRAAGALLLALLLHGRLLA
Transforming growth factor beta -2	TGFB2	19	*Homo sapiens*	P61812	MHYCVLSAFLILHLVTVAL
Serine protease inhibitor Kazal –type4	SPINK4	26	*Homo sapiens*	O60575	MAVRQWVIALALAALLVVDREVPVAA
C -type natriuretic peptide	NPPC	23	*Homo sapiens*	P23582	MHLSQLLACALLLTLLSLRPSEA
Tuberoinfundibular peptide of 39 residues	PTH2	30	*Homo sapiens*	Q96A98	METRQVSRSPRVRLLLLLLLLLVVPWGVRT
Pro-neuropeptide Y	NPY	28	*Homo sapiens*	P01303	MLGNKRLGLSGLTLALSLLVCLGALAEA
Interleukin -8	CXCL8	20	*Homo sapiens*	P10145	MTSKLAVALLAAFLISAALC
Alpha -1-antitrypsin	SERPINA1	24	*Homo sapiens*	P01009	MPSSVSWGILLLAGLCCLVPVSLA
Gastrin -releasing peptide	GRP	23	*Homo sapiens*	P07492	MRGSELPLVLLALVLCLAPRGRA
Plasminogen	PLG	19	*Homo sapiens*	P00747	MEHKEVVLLLLLFLKSGQG
Transforming growth factor beta -3	TGFB3	20	*Homo sapiens*	P10600	MKMHLQRALVVLALLNFATV
Guanylate cyclase activator 2B	GUCA2B	26	*Homo sapiens*	Q16661	MGCRAASGLLPGVAVVLLLLLQSTQS

###  In silico prevision of signal peptide and prediction of h, c and n regions

 SignalP software version 4.1 (http://www.cbs.dtu.dk/services/SignalP-4.1/) was used for the prediction of signal peptides and their sites of cleavage based on the combination of different artificial neural networks.^11^ SignalP online software version 3.0 was (http://www.cbs.dtu.dk/services/SignalP-3.0/) employed for predicting n, h, and c regions of signal peptides. For this purpose, signal peptides were added to the somatropin sequence and analyzed by the program.

###  Analysis of physicochemical features of signal peptides

 The ProtParam program was used to evaluate the physicochemical features of the signal peptides including, theoretical pI, amino acid composition, negatively and positively charged amino acids, grand average of hydropathicity (GRAVY), instability index, aliphatic index, and molecular weight.

###  Analysis of protein solubility 

 SOLpro tool predicts the solubility of a protein upon expression in *E. coli* based on characteristics of primary sequences. Therefore, the SOLpro at http://scratch.proteomics.ics.uci.edu/, was used to determine the protein solubility in *E. coli. *SOLpro tool has a prediction accuracy of above 74%.

###  Prediction of protein localization 

 ProtComp B server, from Softberry, Inc (http://www.softberry.com), was applied for prediction of somatropin destination in connection with various signal peptides. It accomplishes this job using a composition of sequence homology and neural networks.^12^

###  Prediction of the type of signal peptides and cleavage probability

 In prokaryotes, there are three types of signal peptides, including Sec pathway cleaved by either SPase I (Sec/SPI) or SPase II (Sec/SPII), and Tat pathway cleaved by Tat/SPI.^13^ SignalP5.0 server was used for discrimination of three types of signal peptides.^14^ SignalP 5.0 predicts the type of signal peptides based on a deep convolutional and recurrent neural network architecture.^15^ The cleavage probability was also determined by SignalP 5.0 program.

## Results and Discussion

###  In silico prediction of signal peptide and determination of c, h, and n regions 

 SignalP 4.1 was applied for prediction of the most suitable signal peptide for somatropin, enabling its secretion into the periplasmic space in *E. coli.* SignalP 4.1 identifies a signal peptide based on a discriminating score, D-score. The output was tabulated in Table 2, containing five scores of D, C, S, Y, S-mean including cleavage sites and c, h and n regions of signal peptides.

**Table 2:** Signal peptide probability and c, h and n regions

**Signal peptides**	**n-region**	**h-region**	**c-region**	**Cleavage site**	**cleavage probability**	**C-score**	**Y- score**	**S-score**	**S-mean**	**D-score**
appA	4	12	7	AFA	0.9807	0.801	0.786	0.938	0.808	0.797
ccmH	3	9	7	ALA	0.9806	0.773	0.568	0.655	0.472	0.532s
cexE	4	8	7	AIA	0.995	0.691	0.551	0.665	0.504	0.534
cysP	9	9	6	VQA	0.999	0.757	0.770	0.896	0.821	0.794
draA	4	10	7	AHA	0.990	0.717	0.807	0.971	0.921	0.860
dsbD	4	9	7	VFA	0.9705	0.829	0.605	0.649	0.503	0.567
dsbG	4	9	7	AFA	0.9021	0.417	0.447	0.712	0.536	0.480
faeG	5	10	7	AHA	0.9921	0.762	0.814	0.970	0.891	0.851
fecB	6	9	7	AFA	0.9354	0.601	0.424	0.514	0.355	0.398
fedA	5	9	8	AMA	0.9844	0.739	0.815	0.972	0.911	0.860
FimF41a	5	11	7	VMA	0.9827	0.873	0.869	0.978	0.896	0.882
flgI	5	9	7	AQA	0.9692	0.824	0.880	0.981	0.937	0.907
hofQ	4	8	7	VQA	0.9938	0.643	0.474	0.436	0.357	0.430
lolA	5	10	7	VWA	0.9948	0.715	0.675	0.874	0.724	0.693
lptA	11	9	7	AFA	0.9840	0.801	0.711	0.905	0.753	0.726
malE	8	10	9	ALA	0.9270	0.718	0.810	0.988	0.924	0.863
mepA	4	9	7	SLA	0.9500	0.790	0.726	0.860	0.717	0.722
nikA	7	9	7	VHA	0.9155	0.740	0.604	0.710	0.563	0.589
nrfA	10	10	7	VHA	0.9611	0.549	0.408	0.514	0.369	0.394
ompA	4	10	7	AQA	0.9814	0.800	0.841	0.968	0.891	0.865
ompP	6	10	6	ASA	0.8765	0.618	0.649	0.870	0.740	0.692
ompW	5	10	7	AFA	0.9924	0.808	0.863	0.966	0.923	0.891
papK	5	10	7	AIA	0.9415	0.721	0.642	0.837	0.659	0.648
pbpG	6	12	7	AVA	0.9542	0.681	0.753	0.985	0.890	0.817
PelB	6	10	6	AMA	0.9905	0.792	0.875	0.981	0.949	0.910
phoA	5	9	7	TKA	0.9648	0.496	0.613	0.845	0.722	0.688
phoE	5	10	6	VQA	0.9875	0.761	0.807	0.948	0.855	0.829
prsK	5	10	7	AMA	0.9805	0.837	0.854	0.950	0.881	0.867
pspE	4	9	7	VFA	0.9743	0.811	0.593	0.687	0.514	0.564
ptrA	8	9	7	SQA	0.9750	0.699	0.579	0.582	0.504	0.522
sfaS	5	11	7	AQA	0.9551	0.695	0.763	0.961	0.841	0.800
tauA	7	9	7	AQA	0.9441	0.832	0.820	0.947	0.834	0.827
thiB	4	8	6	VFA	0.9667	0.611	0.757	0.962	0.927	0.837
TorT	3	9	6	AFS	0.8362	0.435	0.413	0.593	0.442	0.424
TorA	18	15	7	ATA	0.9628	0.259	0.211	0.286	0.202	0.208
ugpB	7	10	7	AQA	0.9861	0.826	0.821	0.924	0.830	0.825
xylF	6	11	7	AHA	0.9446	0.726	0.806	0.973	0.903	0.851
yfeK	4	10	6	VYA	0.9878	0.711	0.490	0.571	0.398	0.456
yhcN	6	10	7	AFA	0.9780	0.714	0.596	0.793	0.602	0.598
yncJ	5	11	7	LMA	0.8738	0.798	0.851	0.962	0.904	0.876
ynfB	10	12	7	VHA	0.9723	0.819	0.623	0.789	0.590	0.611
zraP	7	12	8	AFA	0.9535	0.786	0.838	0.994	0.929	0.881
ampC	4	10	6	TAS-CS.	0.6246	0.788	0.848	0.942	0.910	0.877
eltB	6	9	7	AHG	0.6339	0.647	0.747	0.954	0.874	0.807
pilC	5	11	7	TNA-SF.	0.8309	0.171	0.392	0.973	0.909	0.635
pcoB	7	10	7	SIA	0.9063	0.369	0.378	0.585	0.449	0.404
eatA	37	13	7	-	-	0.230	0.166	0.329	0.286	0.210
HBP	34	12	7	SLA	0.6063	0.243	0.179	0.262	0.168	0.175
DsbA	4	10	6	ASA-	0.9419	0.572	0.616	0.837	0.717	0.654
Hgh	7	12	6	GSA	0.8990	0.200	0.237	0.539	0.318	0.267
OmpC	5	10	7	ANA	0.9648	0.827	0.863	0.973	0.918	0.889
STII	5	12	7	AYA	0.9604	0.856	0.856	0.971	0.892	0.873
ansB	7	9	7	ALA	0.9587	0.838	0.644	0.707	0.555	0.611
sfmC	7	10	7	AHA	0.9601	0.806	0.595	0.576	0.439	0.537
ompf	6	10	7	ANA	0.981	0.839	0.862	0.946	0.902	0.880
ompt	5	9	7	SFA	0.9250	0.293	0.335	0.538	0.414	0.364
LPP	6	9	5	LLA-GF	0.4598	0.145	0.214	0.581	0.472	0.309
lamB	9	10	7	AMA	0.8549	0.785	0.819	0.981	0.894	0.854
bla	7	10	7	VFA	0.9203	0.624	0.413	0.465	0.334	0.384
mglB	5	12	7	AHA	0.9717	0.767	0.834	0.986	0.923	0.876
Sta1	4	9	7	SFS	0.8744	0.492	0.664	0.939	0.888	0.769
araF	6	11	7	AMA	0.987	0.804	0.844	0.958	0.874	0.858
nmpc	5	12	7	AQA	0.9833	0.835	0.876	0.981	0.930	0.902
ppiA	5	13	7	AMA	0.9564	0.785	0.846	0.989	0.939	0.890
yaaI	6	11	7	AQA	0.7641	0.721	0.806	0.957	0.913	0.856
yhcF	6	8	7	AQA	0.9636	0.737	0.748	0.897	0.777	0.761
yfcQ	4	8	7	AHA	0.9790	0.712	0.783	0.932	0.854	0.816
EfeO	9	11	7	ANA	0.9450	0.585	0.705	0.973	0.875	0.785
glnH	6	10	7	SHA	0.9779	0.740	0.814	0.965	0.910	0.859
rna	7	10	7	ANA	0.9760	0.784	0.835	0.975	0.912	0.871
DsbC	4	10	7	AQA	0.9809	0.764	0.825	0.971	0.898	0.859
rbsB	6	12	8	AMA	0.6795	0.798	0.818	0.979	0.893	0.854
bcsB	6	11	9	TQA	0.8993	0.455	0.615	0.985	0.889	0.744
gfcA	5	10	7	AFA	0.9834	0.8441	0.882	0.985	0.925	0.902
PRH1	6	10	7	RGA	0.542	0.195	0.324	0.657	0.553	0.409
LEAP2	6	10	7	LLG	0.324	0.139	0.165	0.359	0.281	0.208
C10orf99	4	12	7	IFS	0.593	0.255	0.298	0.499	0.384	0.329
PRLH	6	10	7	RGA	0.542	0.195	0.324	0.657	0.553	0.409
HSPG2	5	10	7	LLA	0.986	0.330	0.269	0.356	0.246	0.260
TGFB2	3	10	7	PLS	0.049	0.129	0.186	0.443	0.355	0.248
SPINK4	6	12	8	-	0.051	0.143	0.190	0.453	0.322	0.239
NPPC	6	11	7	SEA	0.9791	0.398	0.566	0.889	0.804	0.678
PTH2	15	9	7	VRT	0.5430	0.156	0.172	0.354	0.310	0.233
NPY	7	13	7	AEA	0.6235	0.578	0.465	0.504	0.413	0.446
CXCL8	5	16	7	ALC	0.5500	0.343	0.420	0.816	0.603	0.488
SERPINA1	7	11	7	SLA	0.8489	0.402	0.289	0.395	0.260	0.278
GRP	6	11	7	GRA	0.8903	0.268	0.242	0.381	0.244	0.243
PLG	-	-	-	GQG	0.4277	0.207	0.239	0.444	0.246	0.242
TGFB3	7	10	10	-	0.147	0.146	0.224	0.610	0.519	0.333
GUCA2B	5	15	7	TQS	0.7021	0.320	0.249	0.369	0.270	0.257

 Thirty-six signal peptides were deleted from further analysis because the D-scores of them were less than the cut off value of 0.570, indicating that they are not efficient for the secretion of somatropin protein.

 Among the analyzed 90 signal peptides, four signal peptides, including pelB, flgl, nmpc, and, gfcA showed the highest D-score value of 0.910, 0.907, 0.902, and 0.902, respectively. Moreover, the results demonstrated that pelB and NPPC have the highest D-score in prokaryotic and eukaryotic expression systems, respectively. Additionally, the lowest scores belonged to HBP and LEAP2 (0.175, 0.208) in prokaryotic and eukaryotic expression systems, respectively.

###  Physico-chemical features of signal peptides 

 Several physicochemical features of 55 remaining signal peptides containing, theoretical pI length, molecular weight, net positive charge, grand average of hydropathicity (GRAVY), instability index and aliphatic index were evaluated by ProtParam server (Table 3). The results showed that the length of signal peptides was between 18 and 28 residues. The results of in silico analysis revealed that the highest molecular weight pertained to ynfB, bcsB, lptA, and efeO (2948.71, 2853.53, 2849.47, and 2845.33 daltons, respectively).

**Table 3:** The physicochemical characteristics of the signal peptides that were analyzed in the study.

**Signal peptides**	**Length**	**M.W. (Da)**	**P.I.**	**Net positive charge**	**GRAVY**	**Aliphatic** **index**	**Instability** **(Separately)**	Instability with hGH*	Stability*	**Solubility**
appA	22	2384.99	8.5	0.9	1.405	155.45	53.16	42.9	u	0.782
cysP	25	2575.15	10	2.1	1.064	164.00	11.14	37.38	S	0.765
draA	21	2135.63	10	2.1	1.162	98.10	16.49	38.41	S	0.885
faeG	21	2027.47	10	2.1	1.005	112.38	11.36	37.90	S	0.883
fedA	21	2231.76	11	1.9	1.290	102.38	29.55	39.70	S	0.869
FimF41a	22	2090.57	10	1.9	1.355	124.55	15.15	38.17	S	0.863
flgI	20	2116.67	8.5	0.9	1.935	185.50	10.64	37.96	S	0.806
lolA	21	2192.70	9.3	0.9	1.324	139.52	16.67	38.43	S	0.764
lptA	27	2849.47	10.3	2.9	0.881	130.37	17.32	37.91	S	0.831
malE	26	2698.34	11.1	2.9	1.012	113.08	2.85	36.27	S	0.879
mepA	19	1887.31	8.5	0.9	1.479	164.74	32.07	40.03	u	0.833
nikA	22	2434.99	10.3	0.9	1.350	137.73	60.45	42.85	u	0.790
ompA	21	2046.50	10	1.9	1.295	121.43	9.52	37.72	S	0.857
ompP	23	2406.88	5.7	1.9	0.904	114.78	44.47	41.21	u	0.798
ompW	21	2093.55	10	1.9	1.210	125.71	1.44	36.92	S	0.824
papK	21	2047.48	8.5	1.9	1.390	140.00	-2.60	36.52	S	0.849
pbpG	25	2705.36	11	1.9	1.228	117.20	57.99	42.81	u	0.800
PelB	22	2228.78	8.3	0.9	1.191	138.18	41.42	40.88	u	0.802
phoA	21	2256.82	10	0.9	0.971	139.52	56.02	42.33	u	0.769
phoE	21	2104.59	10	0.9	1.195	130.00	1.44	36.92	S	0.834
prsK	21	2065.52	8.5	0.9	1.267	121.43	3.27	37.10	S	0.859
sfaS	22	2290.85	9.3	0.9	1.314	146.82	5.41	37.16	S	0.844
tauA	22	2308.72	9.5	0.9	1.055	120.45	34.41	40.16	u	0.824
thiB	18	1974.60	8.8	0.9	1.589	157.22	65.64	42.96	u	0.608
ugpB	23	2342.80	8.3	0.9	0.622	110.87	18.01	38.37	S	0.844
xylF	23	2482.08	9.3	0.9	1.083	161.30	33.61	40.04	u	0.781
yhcN	22	2254.76	10	0.9	1.418	128.64	-2.03	36.39	S	0.764
yncJ	22	2344.91	7.9	0.9	1.541	128.64	15.15	38.17	S	0.795
ynfB	28	2948.71	10	0.9	1.239	163.93	29.32	39.35	S	0.774
zraP	26	2733.37	11.1	0.9	0.746	79.23	28.75	39.37	S	0.834
ampC	19	2022.46	7.8	0.9	1.342	97.89	25.22	39.41	u	0.783
eltB	21	2342.84	9.1	0.9	0.890	111.43	31.10	39.86	S	0.803
pilC	23	2400.92	10	0.9	1.104	110.43	1.01	36.54	S	0.794
DsbA	19	1990.48	10	0.9	1.416	144.21	11.50	38.17	S	0.842
OmpC	21	2078.63	10	0.9	1.552	171.90	14.37	38.20	S	0.797
STII	23	2552.09	9.7	1.9	1.026	102.17	32.43	39.92	S	0.861
ansB	22	2274.76	8.3	1.9	1.136	93.64	-1.15	36.48	S	0.846
ompF	22	2266.83	11	1.9	1.259	150.91	67.18	43.54	u	0.876
sta1	19	2159.72	10	1.9	1.368	123.16	25.28	39.41	S	0.841
lamB	25	2545.22	11	1.9	1.332	125.20	42.97	41.07	u	0.889
mglB	23	2362.89	10	1.9	0.952	102.17	14.15	37.95	S	0.865
araF	23	2348.87	10	1.9	0.878	93.91	96.71	46.83	u	0.876
nmpc	23	2292.84	10	1.9	1.243	119.13	30.34	39.69	S	0.883
ppiA	24	2371.90	8.5	1.9	1.438	98.33	39.94	40.72	u	0.841
yaaI	23	2389.93	7.8	1.9	1.365	114.78	23.74	38.98	S	0.842
yhcF	20	2084.48	8.5	1.9	0.915	98.00	25.79	39.39	S	0.860
yfcQ	18	1962.40	9.5	1.9	1.006	119.44	13.91	38.50	S	0.792
efeO	26	2845.33	12	1.9	0.654	94.23	54.20	42.42	u	0.865
glnH	22	2244.72	10	1.9	1.209	133.18	10.58	37.70	S	0.846
rna	23	2478.94	11	1.9	0.757	106.52	40.05	40.74	u	0.809
DsbC	20	2179.67	10	1.9	1.000	78.50	5.25	37.45	S	0.836
rbsB	25	2494.02	10	1.9	0.948	109.60	11.14	37.38	S	0.879
bcsB	25	2853.53	10	1.9	0.688	58.80	48.06	41.66	u	0.874
gfcA	21	2293.87	10	1.9	1.019	98.10	40.98	40.83	u	0.842
NPPC	23	2494.05	6.5	1.9	1.07	165.65	95.44	46.69	u	0.737

*S = Stable, U = Unstable *The proteins whose instability index was higher than 40 were predicted as unstable and the values under 40 might be stable.

 The most hgigh GRAVY values were belonged to signal peptides flgI, thiB, OmpC and yncJ (1.935, 1.589, 1.552, and 1.541, respectively). The highest aliphatic index scores belonged to flgl, ompC, NPPC, mepA, and cysP (185.50, 171.90, 165.65, 164.74, and 164.00, respectively)

 Another evaluated physicochemical feature of signal peptides was the instability index. The results demonstrated that papK, yhcN, ansB, and pilC (-2.60, -2.03, -1.15, and 1.01, respectively) were the most stable signal peptides, separately and in connection with somatropin. The proteins whose instability index was higher than 40 were predicted as unstable, and the values under 40 might be stable.

###  Prediction of protein solubility 

 The results of somatropin solubility in fusion with various signal peptides have shown in Table 3. The results demonstrated that the highest solubility were belonged to lamb, draA, faeG, nmpc, rbsB, and malE signal peptides (0.889, 0.885, 0.883, 0.883, 0.879, and 0.879, respectively).

###  Prediction of the protein localization

 The analysis results for sub-cellular localization by ProtCompB server indicated that the final localization sites were the outer membrane, inner membrane, and periplasmic space for 13, 15, and 18 signal peptides, respectively. Furthermore, analysis for the final localization of somatropin with signal peptides faeG, FimF41a, ompA, papK, prsK, lamb, nmpc, bcsB, and gfcA revealed that somatropin could be secreted by these signal peptides (Table 4).

**Table 4:** Analysis of secretion pathways and final localization of human somatropin mediated by different signal peptides

**Signal peptides**	**Secretion pathway**	**Reliability score**	**Cytoplasmic**	**Membrane**	**Secreted**	**Periplasmic**	**Final prediction site**
appA	Sec/SPI	0.9925	1.68	4.70	0.00	3.62	Inner membrane
cysP	Sec/SPI	0.9795	1.42	6.26	0.00	2.33	Outer Membrane
draA	Sec/SPI	0.9984	0.86	4.74	0.48	3.92	Outer Membrane
faeG	Sec/SPI	0.9984	0.53	1.75	5.03	2.69	Extracellular
fedA	Sec/SPI	0.9963	0.32	7.13	2.55	0.00	Inner Membrane
FimF41a	Sec/SPI	0.9963	0.00	2.40	6.31	1.29	Extracellular
flgI	Sec/SPI	0.9892	1.09	5.84	0.00	3.07	Inner Membrane
lolA	Sec/SPI	0.9975	0.43	2.34	0.00	7.23	periplasmic
lptA	Sec/SPI	0.9846	0.55	6.03	0.00	3.42	Outer Membrane
malE	Sec/SPI	0.9909	0.71	3.44	0.00	5.85	Periplasmic
mepA	Sec/SPI	0.9925	0.58	7.14	0.00	2.29	Outer Membrane
nikA	Sec/SPI	0.9001	0.8	5.47	0.00	3.73	Inner membrane
ompA	Sec/SPI	0.9977	0.13	1.07	5.21	3.58	Extracellular
ompP	Sec/SPI	0.9834	1.76	7.82	0.00	0.42	Outer membrane
ompW	Sec/SPI	0.9965	0.00	6.16	2.12	1.72	Outer Membrane
papK	Sec/SPI	0.978	0.11	1.83	7.41	0.65	Extracellular
pbpG	Sec/SPI	0.9844	0.64	2.43	0.00	6.93	Periplasmic
PelB	Sec/SPI	0.9967	1.29	1.42	3.33	3.96	Periplasmic
phoA	Sec/SPI	0.9924	1.15	7.68	0.00	1.17	Inner membrane
phoE	Sec/SPI	0.9973	0.28	8.63	0.43	0.66	Inner Membrane
prsK	Sec/SPI	0.9929	0.00	2.13	6.21	1.66	Extracellular
sfaS	Sec/SPI	0.9831	1.52	3.49	0.00	4.99	Periplasmic
tauA	Sec/SPI	0.9096	0.74	5.50	0.00	3.75	Outer Membrane
thiB	Sec/SPI	0.9867	0.80	2.85	0.00	6.35	Periplasmic
ugpB	Sec/SPI	0.995	0.55	3.17	0.00	6.29	Periplasmic
xylF	Sec/SPI	0.9969	1.40	3.81	0.00	4.80	periplasmic
yhcN	Sec/SPI	0.9896	0.26	8.20	1.54	0.00	Inner membrane
yncJ	Sec/SPI	0.9078	1.21	7.34	0.00	1.45	Inner membrane
ynfB	Sec/SPI	0.9881	0.00	2.65	0.98	6.37	periplasmic
zraP	Sec/SPI	0.9931	0.57	2.46	0.00	6.97	Periplasmic
ampC	Sec/SPII	0.6243	0.93	2.63	0.00	6.39	Periplasmic
eltB	Sec/SPI	0.7337	0.97	7.60	0.00	1.43	Outer membrane
pilC	Sec/SPI	0.9545	0.99	8.63	0.29	0.10	Outer membrane
DsbA	Sec/SPI	0.9875	0.00	8.44	0.68	0.89	Inner membrane
OmpC	Sec/SPI	0.9874	0.33	6.55	1.58	1.54	Inner membrane
STII	Sec/SPI	0.9953	0.11	8.42	1.47	0.00	Outer membrane
ansB	Sec/SPI	0.9641	0.60	6.46	0.00	2.94	Inner membrane
ompF	Sec/SPI	0.9896	0.62	8.19	0.74	0.45	Inner membrane
sta1	Sec/SPI	0.9672	0.08	9.51	0.41	0.00	Inner membrane
lamB	Sec/SPI	0.9865	0.32	3.71	3.88	2.09	Secreted
mglB	Sec/SPI	0.9971	0.80	5.63	0.00	3.57	Inner membrane
araF	Sec/SPI	0.9941	0.22	3.73	0.00	6.05	Periplasmic
nmpc	Sec/SPI	0.9964	0.00	0.96	7.84	1.20	Secreted
ppiA	Sec/SPI	0.9934	0.54	5.45	0.00	4.01	Outer membrane
yaaI	Sec/SPI	0.78	0.18	4.43	2.80	2.59	Inner membrane
yhcF	Sec/SPI	0.9801	0.86	8.13	0.00	1.01	Outer membrane
yfcQ	Sec/SPI	0.9956	1.58	7.04	0.37	1.01	Inner membrane
efeO	TAT	0.5377	0.25	0.49	0.00	9.26	Periplasmic
glnH	Sec/SPI	0.9959	0.18	3.97	0.00	5.85	Periplasmic
rna	Sec/SPI	0.9914	0.75	8.88	0.37	0.00	Outer membrane
Dsbc	Sec/SPI	0.9955	0.46	5.80	0.00	3.75	Inner membrane
rbsB	Sec/SPI	0.9969	0.00	2.61	2.76	4.63	Periplasmic
bcsB	Sec/SPI	0.9793	0.02	2.28	7.17	0.53	Secreted
gfcA	Sec/SPI	0.9959	0.19	2.21	6.76	0.85	Secreted
NPPC	Sec/SPI	0.9877	1.33	7.54	0.00	1.12	Inner membrane

###  Prediction of cleavage probability and the type of signal peptides 

 The remaining 55 signal peptides were examined for their secretory pathway(s) by using signal P5.0 software. The results showed that except efeO (TAT pathway) and ampC (sec/SPII), all of these signal peptides were specific for the Sec/SPI pathway (Table 4). The cleavage probability of each signal peptides was tabulated in Table 2.

###  Selection of appropriate signal peptide

 First, the signal peptides with final localization in periplasmic space was selected and sorted according to the aliphatic index. Then, the stability and solubility of target protein in connection with the selected signals was examined. The signal peptides with which somatropin remained stable and soluble were selected as the appropriate peptide signal (Table 5).

**Table 5:** Characteristics of most efficient signal peptides for periplasmic expression of human somatropin based on their determinant features

**Signal peptides**	**Aliphatic index**	**Gravy**	**D-score**	**Stability**	**Solubility**
ynfB	163.93	1.239	0.611	39.35	0.774
xylF	161.30	1.083	0.851	40.04	0.781
thiB	157.22	1.589	0.837	42.96	0.608
sfaS	146.82	1.314	0.800	37.16	0.844
lolA	139.52	1.324	0.693	38.43	0.764
PelB	138.18	1.191	0.910	40.88	0.802
glnH	133.18	1.209	0.859	37.70	0.846
pbpG	117.20	1.228	0.817	42.81	0.800
malE	113.08	1.012	0.863	36.27	0.879
ugpB	110.87	0.622	0.825	38.37	0.844
rbsB	109.60	0.948	0.854	37.38	0.879
ampC	97.89	1.342	0.877	39.41	0.783
efeO	94.23	0.654	0.785	42.42	0.865
araF	93.91	0.878	0.858	46.83	0.876
zraP	79.23	0.746	0.881	39.37	0.834

*E. coli* is the economical and straightforward host for the expression of recombinant proteins.^16^ However, overexpression of recombinant proteins in the intracellular space of *E. coli* is usually associated with insoluble aggregate and inclusion body formation. To keep appropriate folding, the proteins should be avoided from the reductive environment of the cytoplasm. Hence, the secretory expression has several advantages for the production of recombinant proteins, compared with cytosolic systems.

 The secretion of the target protein requires transporting across the cytoplasmic membrane. In bacteria, Sec, SRP, and TAT are three major protein secretion pathways for the carriage of proteins through the plasma membrane. These protein transport systems depend on the presence of suitable signal peptides on proteins. Signal peptides are short amino terminal peptides that affect the biosynthesis, folding, and stability of the corresponding target proteins.^17^ Although various signal peptides differ in their sequences, they share conserved physicochemical properties, including aliphatic index, molecular weight, instability index, Gravy, net positive charge, and theoretical pI. The three important regions of signal peptides include an amino terminal positively-charged region, a hydrophobic central region, and a carboxyl-terminal polar region that contains the cleavage site (a conserved A-X-A motif). It has demonstrated that the n region in the signal peptide has an essential role in the primary phase of protein secretion across the membranes.^18^ Also the n-region responsible for the net positive charge of the signal peptide. In addition, the presence of the basic residues in this region may be indispensable for the performance of an efficient signal peptide.^19^

 Further to the charge of the n-region, the c-region has an intense effect on the performance of membrane transport by both the Tat and Sec pathways. The third region of signal peptides that can affect the secretion output is the hydrophobic helical H region of the signal peptides. Also, the central h-region of signal peptides are important because the length and hydrophobic density of h-region intensify the hydrophobicity levels and facilitate the protein secretion.^19,20^

 In the present study, the physicochemical features of the 90 signal peptides were analyzed for secretory expression of somatropin in *E. coli*.

 As shown in Table 3, flgI, OmpC, NPPC, mepA, and cysP showed the highest hydrophobicity levels (185.50, 171.90, 165.65, 164.74 and 164.00, respectively) among the studied signal peptides whereas, the signal peptides, bcsB, DsbC, zraP, ansB, and araF showed the lowest hydrophobicity (58.80, 78.50, 79.23, 93.64, and 93.91, respectively). Previous studies reported that OmpC has the highest aliphatic index, which is in agreement with our results.^21^

 Analysis for secretory pathway revealed that all 55 Signal peptides (except efeO) are specific for the Sec pathway with reliability scores of more than 0.9 (Table 4). Therefore, our findings were consistent with some previous reports.^9,22^ Sec exportome polypeptides have a cleavable, Sec-specific, n-terminal signal peptides that translocates proteins across the inner membrane (I.M.) in an unfolded state.^23,24^

 There are two methods for selecting a signal peptide for any given protein, including experimental / trial and error method, and in silico analysis method. The advantages of using a bioinformatics program before starting an experimental study are increasing the precision and validity and reducing experimental research expenses.

 In this study, online bioinformatic tools were used to find suitable signal peptides for periplasmic expression of recombinant somatropin in *E. coli*. Different signal peptides, including 17 eukaryotic and 73 prokaryotic signal peptides, were evaluated. The D-score parameter was used to determine an appropriate signal peptides. D-score is also used to sort signal peptides in the first step. According to the D-scores (Table 2), 55 out of 90 selected signal peptides were identified as signal peptides for somatropin. Data were sorted based on the priority of D-scores, final localization, h-region length, aliphatic index, Gravy, and solubility, respectively (Table 5). According to this sorting, pelB, flgl, nmpC, GfcA, OmpW, PpiA, and OmpC showed the highest D-score. However, pelB and OmpC showed the highest D-score in other bioinformatics studies.^21^ The results of analysis revealed that somatropin in connection with 34 signal peptides was stable and directed toward the Sec pathway, 9 signal peptides mediated the secretion, and 15 signal peptide translocated the somatropin into the periplasmic space.

 Zamani et al analyzed the secretion of somatropin by L-asparaginase II signal sequence and reported that successful secretion of somatropin is not achieved using the L-asparaginase II signal sequence.^22^

 The expression of somatropin with the NPR, STII and DsbA signal peptides using RRI as the host cell, showed that the DsbA was the most effective signal peptide for somatropin gene with 80% higher expression level compared to the reference vector.^23^

 Previous studies^25^ demonstrated the high secretion of somatropin with phoA signal peptide, but in our research, phoA was not the right candidate due to lower D-sore (0.688) and final localization in the inner membrane.

 This study evaluated 90 different signal peptide to find the most applicable signal peptide for secreting the recombinant somatropin protein in the *E. coli*. The results of the present study showed that ynfB, sfaS, lolA, glnH, and malE has all the features needed to be selected as suitable signal peptides for somatropin protein

## Conclusion

 In this research, various signal peptides were appraised for the periplasmic expression of somatropin in *E. coli*. The selection was based on the combination of hydrophobicity, D score, solubility, stability, and the final localization.

 The results indicated that specific signal peptides, including ynfB, sfaS, lolA, glnH, and malE have the highest scores and could be used for soluble periplasmic expression of somatropin in *E. coli*. However, the proof of these results should be verified by an experimental study.

## Acknowledgments

 This study was supported by National Institute for Medical Research Development(NIMAD)grant no. 958751.

## Competing Interests

 The authors have no conflict of interest to declare.

## Ethical Approval

 This research was approved by Iran National Committee for ethics in Biomedical Research (958751).
